# Experiences and perceptions of people with headache: a qualitative study

**DOI:** 10.1186/1471-2296-7-27

**Published:** 2006-05-02

**Authors:** Deborah A Leiper, Alison M Elliott, Philip C Hannaford

**Affiliations:** 1 Aberdeen Royal Infirmary, Westburn Road, Foresterhill, Aberdeen, AB25 2ZN, Scotland, UK; 2 Department of General Practice and Primary Care, University of Aberdeen, Foresterhill Health Centre, Westburn Road, Aberdeen, AB25 2AY, Scotland, UK

## Abstract

**Background:**

Few qualitative studies of headache have been conducted and as a result we have little in-depth understanding of the experiences and perceptions of people with headache. The aim of this paper was to explore the perceptions and experiences of individuals with headache and their experiences of associated healthcare and treatment.

**Methods:**

A qualitative study of individuals with headache, sampled from a population-based study of chronic pain was conducted in the North-East of Scotland, UK. Seventeen semi-structured interviews were conducted with adults aged 65 or less. Interviews were analysed using the Framework approach utilising thematic analysis.

**Results:**

Almost every participant reported that they were unable to function fully as a result of the nature and unpredictability of their headaches and this had caused disruption to their work, family life and social activities. Many also reported a negative impact on mood including feeling depressed, aggressive or embarrassed. Most participants had formed their own ideas about different aspects of their headache and several had searched for, or were seeking, increased understanding of their headache from a variety of sources. Many participants reported that their headaches caused them constant worry and anguish, and they were concerned that there was a serious underlying cause. A variety of methods were being used to manage headaches including conventional medication, complementary therapies and self-developed management techniques. Problems associated with all of these management strategies emerged.

**Conclusion:**

Headache has wide-ranging adverse effects on individuals and is often accompanied by considerable worry. The development of new interventions or educational strategies aimed at reducing the burden of the disorder and associated anxiety are needed.

## Background

Headaches are common, with almost everyone experiencing at least one during their lifetime [1]. The lifetime prevalence of headache has been estimated to be up to 96% in the adult population [1], and one-year prevalence estimates range from 38% [2] to 68% [3]. Many people experience headaches on a frequent basis: a recent UK survey found that 18% of respondents had experienced headaches one to three times a week during the previous three months, with 6% experiencing them more often [4]. Headaches have been shown to have an adverse impact on work, [4-7] social and family life [8-11] and are associated with large medical costs [12]. Studies investigating the management of headache have shown that most individuals use prescription and/or non-prescription (over-the-counter) medication [5,9]. Many individuals manage their headaches without consulting their general practitioners [5,9,13], some choosing alternative forms of healthcare.

Much of our knowledge about the frequency and management of headache have come from quantitative studies. While these studies provide important information about the magnitude and impact of the problem, they are not able to examine unique aspects about the condition for individual patients. By contrast few qualitative studies of headache have been conducted and as a result we have little in-depth understanding of the experiences and perceptions of people with headache. An in-depth understanding of the experiences of people with headache is important if we are to develop new treatments, interventions or educational strategies that might reduce the burden of the disorder and qualitative studies of headache have been called for to address this gap [14]. Such studies are beginning to emerge, [15-17] but work has tended to focus on migraine and further research is still needed. We report here the findings from a qualitative study conducted to explore the experiences and perceptions of people with headache, and their experiences and expectations of associated healthcare and treatment.

## Methods

### Design and sample

Participants were sampled from respondents to a population-based study [18,19] conducted in the Grampian region of Scotland, UK which investigated the prevalence and natural history of chronic pain (pain or discomfort, present either all the time, or on and off, which had persisted for three months or longer [20]). A total of 660 respondents in 2000 reported having chronic pain in their head. Of these, 73 individuals identified the head as either the only site, or the main site, of their pain and were defined as having chronic head pain. A total of 59 of these individuals indicated a willingness to participate in further research. From this group, a purposive sample of 30 individuals was selected to include males and females aged 65 and under, with different levels of severity of chronic pain (as measured by the chronic pain grade [21]) and self reported use of different healthcare services. The 30 individuals were sent letters detailing the purpose of the study and inviting them to participate in an interview about their headaches. Individuals who did not, or no longer, suffered from headaches were given the opportunity to opt-out if their participation was not felt to be appropriate. Reminders were sent four weeks later to non-respondents. Ethical approval for the study was given in advance by the Grampian Research Ethics Committee.

A semi-structured interview schedule (Table 1) was developed, covering issues identified as potentially important from a review of the literature. The interview started with an open question inviting participants to tell us about their headaches. This allowed the participants to raise any issues that they felt to be important. If issues arose that weren't on the topic guide the interviewer ensured that these issues were discussed further during the interview. The closed questions detailed in the schedule were not directed towards the participants, but simply used as a guide by the interviewer to the topics to be covered and to identify further relevant areas of interest. Open-ended questions were used throughout the interviews to encourage participants to answer openly and unrestrictedly. The schedule was piloted on four researchers with experience in conducting qualitative interviews so that areas for improvement could be identified. This led to some amendments to the schedule. Formal headache diagnoses were not sought from GP records. Participants were asked to self-report what types of headache they suffer from.

**Table 1:** The interview schedule

1. Could you tell me a bit about your headaches? e.g.
• How long have you been suffering from headaches?
• There all the time/on and off?
• Triggers?
• Associated features? (e.g. aura, vision problems)
• Severity, intensity?
2. How would you say your headaches affect your life? e.g.
• Do they stop you from working/carrying out daily duties?
• Do they affect your social life?
• Do they affect your ability to sleep?
• Do they affect your mood?
3. Have you ever been to see your GP about your headaches?
**YES**
• What prompted you to go the first time?
• How did you feel the first consultation went?
• Would you say that you got what you wanted from the consultation?
• What would you say makes a consultation good or bad?
**NO**
• Why haven't you been to see him/her?
• What would prompt you to go?
4. Do you still go to see your GP?
**YES**
• Why do you still go?
• How do you feel the consultations go now?
**NO**
• Why don't you go to see him/her anymore?
How important is it to have a diagnosis?
5. Have you ever seen a hospital specialist about your headaches?
**YES**
• How did/do you feel the consultation goes?
**NO**
• Go to Question 6
6. Have you ever seen an alternative specialist for your headaches?
**YES**
• What prompted you to go the first time?
• Were you satisfied with the consultation?
• Did you get what you wanted from the consultation?
• Do you still see him/her?
• Does your GP know you go?
**NO**
• Would you ever consider going to see an alternative therapist?
7. What types of medications have you used/do you use to relieve the pain?
≺ Prescribed?
≺ Non-prescribed?
≺ Alternative? (If not, would you consider using them?)
Why have you used these?
How helpful were they?
Why do you take medication e.g. to prevent/treat?
8. What else do you do to relieve your head pain?
9. Do you ever use other means such as self-help groups, the Internet or magazines/newspapers for information or help?
10. Do you feel you know enough about your condition?
11. How well do you feel your condition is managed either by a professional or in terms of self-management?
12. How do you view the future with regard your headaches?

The interviews were conducted by DAL between October and December 2002. All of the interviews took place in participants' homes except for one which took place on University premises at the participant's request. Informed consent was sought and granted in all patients interviewed and participants' anonymity and confidentiality were ensured. All interviews were audio-taped with the participants' permission and lasted an average of 45 minutes. Immediately after each interview, brief notes were made by DAL, detailing interesting points made, or behaviours displayed, by the participants. Within two days after the completion of the interview the tapes were transcribed verbatim by the interviewer. A sample of tapes and transcripts were reviewed by AME to check that the interviews had been accurately transcribed. Tapes and transcripts were marked with ID numbers to ensure anonymity and were stored securely to maintain confidentiality.

### Analysis

The interviews were analysed using the Framework approach: a thematic analysis consisting of five stages [22]. The first stage, 'familiarisation', was achieved by reading each transcript and noting down recurring issues. All transcripts were read twice by DAL and once by at least one other member of the research team (AME and PCH). Having done this, the second stage, 'identifying a thematic framework' was completed by arranging the recurrent issues that had emerged into themes and sub themes (Table 2). Interesting and relevant quotes were also identified at this stage to illustrate the themes. Themes and sub-themes were given unique codes, which were marked on the margins of the transcripts to identify interesting issues arising – this process constitutes the third stage, 'indexing'. For the fourth stage, 'charting', charts were constructed for each of the main themes that emerged, with sub-theme categories placed along the top of the charts and participants placed down the sides. By conducting each of these four steps accurately and rigorously, the fifth step, 'mapping and interpretation', was simply achieved by placing relevant sections of the transcript into the appropriate charts. The Framework approach makes between and within subject analysis possible enabling researchers to compare and contrast participants' opinions and perceptions in each sub-theme, therefore illustrating the similarities and differences between them. Reliability of the data and interpretation of the findings were checked at each stage of the process (AME and PCH).

**Table 2:** Themes and sub-themes

Impact on life - inability to function fully - unpredictability of headaches - effect on mood - getting on with things

Knowledge and understanding - lay understanding and explanation - seeking of information - worry

Management - use of conventional medication - use of complementary therapies - GP management - own management techniques

## Results

### Response

Nineteen out of the thirty individuals contacted agreed to participate in the study, 5 respondents indicated that they did not wish to participate, and the remaining 6 did not respond. Reasons for non-participation were not sought. A total of 17 interviews were conducted. Mutually convenient interview times could not be arranged for the last two individuals within the timescale of the study.

### Sample characteristics

Table 3 details the participant characteristics, headache characteristics and associated health care utilisation in the last year of the participants. Most respondents reported suffering from migraines or tension-type headaches. The frequency of headaches in the sample ranged from daily to monthly. Many participants reported that the frequency of their headaches changed depending on the time of year and their current circumstances (i.e. levels of stress at work, family commitments, other health problems etc). Most of the individuals interviewed had been suffering with headaches for several years. Most participants reported that their headaches were of mild to moderate severity, but some reported severe headaches.

**Table 3:** Characteristics of participants

**Participant details**					**Headache characteristics**				**Associated health care utilisation in last year ^§^**						
ID No.	Sex	Age	Educa-tion*	Soc-ec class **	Self-reported type of headache	Frequency of headache	Time patient has had headaches	Severity (1-mild to 4-severe)	Saw GP	Saw HS	Saw PT	Saw AT	Used PM	Used NPM	Used AM

552	M	38	Degree	3M	High BP related	weekly	a few years	1							
2107	F	52	High school	3N	Tension-type	weekly to monthly	12 years	1							
2147	F	36	High school	3N	Tension-type Sinus related	-	5 years	2							
2248	M	50	Degree	2	Tension-type Sinus related	3–4 times a week	30–40 years	1							
2466	F	53	High school	3N	Migraine/tension-type	weekly to monthly	15–20 years	2							
2843	M	46	No qual	3M	Chronic daily	daily	2–3 years	2							
3220	M	48	No qual	3N	Tension-type	daily	many years	4							
4211	F	56	Voca-tional	2	Sinus related	daily	7 years	2							
5316	M	51	High school	2	Migraine	every 2–3 weeks	40 years	1							
5763	M	51	No qual	3M	Tension-type	2–3 times a month	2 years	1							
6317	F	60	No qual	3N	Migraine	Daily to weekly	45 years	1							
6355	F	47	High school	2	Migraine	weekly to monthly	40 years	1							
8795	M	44	High school	3N	Migraine/chronic daily	Daily to weekly	30–40 years/2 years	2							
9029	M	65	Degree	-	Tension-type/eye related	every 4–6 weeks	many years	4							
9423	F	40	Voca-tional	3N	Migraine	twice a month	25 years	3							
9672	M	44	High school	3M	Migraine	weekly	15–20 years	2							
10063	F	50	No qual	4	Migraine	-	20 years	3							

Participant details and associated health care utilisation were collected as part of the postal survey in 2000. Headache characteristics were self-reported at the time of interview except for severity which was assessed using the Chronic Pain Grade during the postal survey in 2000.

^§^Associated health care utilisation in the last year looked at whether participants had visited a general practitioner (GP), hospital specialist (HS), physical therapist (PT) or alternative therapist (AT) and whether they had used prescription medicine (PM), non-prescription medicine (NPM) or alternative medicine (AM).

* Level of education (No qual = no formal education qualification)

** Socio-economic class (1 = highest social class, 5 = lowest social class. M = manual, N = non-manual)

### The themes

A number of interesting issues were raised throughout the interviews, many of which were inter-related and overlapping. However, three broad themes emerged: 'impact on life', 'knowledge and understanding', and 'management'. A summary of the themes and sub-themes emerging from the interviews is shown in Box 2.

#### Impact on life (Table 4)

**Table 4:** Quotes relating to impact on life

Inability to function fully – *"You canna *(can't)*think clearly and you look at a sheet of paper and try and read it and you're not taking it in... your brain doesna *(doesn't)*work as well. You dinna *(don't)*function as well with a migraine." (Patient 6317, Female, 60)*

Inability to function fully – *"... I can't do like the kids need help with their homeworks occasionally, well if Dad's upstairs in a darkened room with a pounding head, well, he's not there for them." (Patient 8795, Male, 44)*

Unpredictability of headaches – *"... if I've been really looking forward to something, excited about something and that, as I say, it arrives, you know, that day's the day that you maybe get a migraine and you just, you're useless, you know, you can't do anything." (Patient 9423, Female, 40)*

Effect on mood – *"... if you've got it every day it just, it starts to drag you down a bit. You get a bit depressed I suppose." (Patient 2843, Male, 46)*

Getting on with things – *"... I try to push my head way to the back and I'm thinking, right, just get on with life, just forget about it. Because I've had it so long now it's just part of me." (Patient 4211, Female, 56)*

Almost every participant reported that various aspects of their lives had been adversely affected by their headaches including work, social and family life and mood.

##### Inability to function fully

Most participants mentioned that it was difficult to carry out daily activities and that their headaches stopped them doing things to the best of their abilities. At work, several people found it hard to concentrate when suffering a headache and felt their performance had been limited. Most had experienced disruption to their work or careers in some way and taking time off was common. Some participants stated that their headaches had prevented them from being promoted and one reported that her constant headaches had not been compatible with her previous work, forcing her to change career. Accounts were also given of several ways in which family and social life was disrupted by an inability to function fully. Some people had been forced to give up hobbies for example reading and hill-walking and common daily activities like driving and sleeping were often affected. Several people spoke of wanting to be alone when suffering from a headache and not being bothered by anything or anyone. This appeared to be particularly problematic for those with more frequent headaches, and some expressed concerns about how this affected their relationships with family and friends.

##### Unpredictability of headaches

Several participants found that their headaches were unpredictable and said that they had a lack of control over the course of a headache which impacted on all aspects of their life. It was common for example for participants to have to be sent home from work because a headache had developed. Some participants reported that the unpredictability of headaches had also impacted on their family lives. For example, many spoke of incidents where their headaches had caused them to miss out on something they were looking forward to or had spoiled their enjoyment of an event. Those who had found effective ways of managing and coping with their pain were less troubled by the fact that their headaches could occur at any time.

##### Effect on mood

The headaches also affected participants' lives by negatively impacting on mood. Descriptions of the effects headaches had included feeling depressed or down, self-pity, aggression and embarrassment. Depression seemed to occur quite commonly among the participants and one man disclosed suicidal feelings because of his constant pain.

##### Getting on with things

In spite of the pain and their lives being adversely disrupted, some participants expressed the need to get on with things, stressing the importance of carrying on and not letting headaches govern them. Some participants went to work and attended social engagements when suffering a headache and spoke of persevering because they felt a responsibility to others. Some had begun to feel that their headaches were just part of their lives. This feeling seemed to be more common among those who had suffered headaches for many years.

#### Knowledge and understanding (Table 5)

**Table 5:** Quotes relating to knowledge and understanding

Lay understanding and explanation – *"And I don't know what the cause of them is and was, I mean, I speculated and thought, could it be this and could it be that... I've been taking HRT and whether it coincided with that... prior to recently I suppose I had considered that they were caused by stress... Stress is a major thing with me, sort of getting very anxious and tense... Hormones was one of the things that I kept tossing about, they weren't migraines*... *I thought maybe it's diet, some of the things that I'm eating that could be triggering these things." (Patient 2107, Female, 52)*

Lay understanding and explanation – *"... I know my first... migraine that I ever had... was on the first day at first year at school... So whether it was because it was a stressful situation, or whether it was because I was starting to develop at that age, I don't know." (Patient 9423, Female, 40)*

Seeking of information – *"It might be the library if I'm on a particular quest for something... Or I might buy a magazine or, you know, any of the journals, nursing things... " (Patient 2466, Female, 53)*

Seeking of information – *"I'd like to know why. You know, I've heard different reasons for it but I haven't heard a proper reason for it. You know, why that, why that causes your headache. It would be nice to know." (Patient 552, Male, 38)*

Worry – *"Well, my friend, well, I suppose it was this chair he sat in and took a brain haemorrhage. My nephew had a brain haemorrhage...and I'm just frightened there's something going on." (Patient 4211, Female, 56)*

Participants had developed their own ideas about aspects of their headaches, often suffered from worry about their condition and were seeking additional information from a variety of sources.

##### Lay understanding and explanation

Most participants had formed their own ideas about different aspects of their headaches including diagnosis, underlying cause and severity. These ideas were often influenced by the experiences of friends and family or from articles they had read. Some participants had not received a formal diagnosis for their headaches but had come up with one themselves based on how their symptoms compared with others they knew or had read about. Patients who believed they had a stressful lifestyle for example diagnosed themselves with tension-type headache, while those with frequent severe headaches often believed they must be suffering from migraines. Several participants had also developed their own ideas on the cause of their headaches and many believed that the underlying cause of their pain was serious. Brain tumours, haemorrhages and strokes were mentioned as potential aetiologies. Many patients had also identified triggers for their headaches such as stress, diet and lifestyle factors. Patients were also trying to understand changes in the severity of their headaches. Reasons given for why headaches had improved included being more relaxed, changing diet, and getting older. Headaches which had deteriorated often caused worry and reinforced the belief of a serious underlying pathology.

##### Seeking of information

Many participants had searched, or were searching, for an increased understanding of their headaches to try to make sense of it all. In particular patients were looking for a diagnosis or explanation of the cause of their headaches. Some participants preferred to obtain advice directly from their general practitioners. GP's were often consulted for information, particularly about new treatments or details of drug side effects or interactions. Many participants reported that they had also read books, magazine articles or searched the Internet for information about headaches. Information was also sought from others such as friends and family. Most participants had obtained information from a variety of sources, and some considered themselves to be quite knowledgeable about their condition as a result.

##### Worry

Several participants said that their headaches had, at some point, caused them constant and often substantial worry. Many had been worried and frightened when they first started suffering headaches, a particular concern being that the underlying cause might be serious. Others spoke of being greatly worried even though they had suffered headaches for years. Interestingly, receipt of a formal diagnosis from a GP did not seem to reduce worry in some participants and had triggered many to search for increased knowledge and understanding of the problem. It also resulted in a number of participants consulting their GP with the expressed wish for further diagnostic tests. The few participants who had received brain scans to rule out serious underlying pathology stated that they had been relieved to receive the "all clear" and their worry had reduced afterwards. Indeed, some reported that their headaches had diminished as a result.

#### Management (Table 6)

**Table 6:** Quotes relating to management

Use of conventional medication – *"... I wonder myself sometimes because I just wonder if some pills is working against the other eens *(ones)*and ken? *(you know)*Cos I'm taking (pauses) I think I'm on about ten different pills a day." (Patient 3220, Male, 49)*

Use of conventional medication – *"... it's (treatment) not as effective as it was when I started using it... I spoke to my doctor about it... it's not as effective as what it was... " (Patient 9423, Female, 40)*

Use of complementary therapies – *"But it's about £30 a session. Which just, it wasn't making a big enough difference to warrant keeping going. So I stopped going there." (Patient 9672, Male, 44)*

GP management – *"... the GP that I see now, she is absolutely, we have a terrific relationship and she has been marvellous, absolutely wonderful. And I probably could talk to her about anything." (Patient 2107, Female, 52)*

GP management – *"There is sometimes I've come out feeling that was a waste of time, they could have done a bit more or taken a bit more interest." (Patient 10063, Female, 50)*

Own management techniques – *"... I had a migraine... I was actually sitting in the bedroom on the floor banging my head off the wall ... it sounds terrible bashing your head off the wall but the initial contact seemed to kill the pain." (Patient 10063, Female, 50)*

Most participants had used or were still using a variety of management strategies including conventional, complementary and personally developed management techniques.

##### Use of conventional medication

Nearly all participants used conventional medication. Participants who reported having migraines most often used drugs obtained by prescription, whereas those who had tension or sinus-related headaches generally reported using non-prescription treatments. Some individuals used medicines obtained by both routes, particularly those who experienced a variety of headache types or who had chronic daily headaches and many found non-prescription medication just as effective as that obtained on prescription. There were several positive comments made about medications, however, a number of individuals expressed concerns. The lack of effectiveness of medications, side effects and drug interactions were of most concern. Many of those who had been on medication for their headaches for a long time no longer found their medicines as effective as they had initially. A number of the participants who reported suffering from side effects also had relatives or friends who had experienced problems while taking medication. Drug interactions appeared to be a problem for a number of respondents particularly for those who took a number of different medicines. A number of people admitted to taking more tablets than they should because their headaches were so severe.

##### Use of complementary therapies

Several participants had used complementary therapies, although only after conventional treatments had been tried first. Homeopathy and reflexology were the most frequently mentioned therapies. Most users were positive about their experiences, and most of the participants who had never tried them found the idea appealing. Many individuals had stopped using alternative therapies, despite finding them useful, because they cost too much while others had stopped using them because they simply didn't find them helpful.

##### G P management

Most participants had consulted, or were still consulting their GP about their headaches. Many were satisfied with their care and generally had positive opinions of their doctors although some negative comments were made including that the GP was dismissive or disinterested in the problem. Reasons for seeking or not seeking help from a GP for headache were explored. Headache severity was a strong influence, with a change in, or worsening of, symptoms often the prompt for consulting. Effectiveness of management strategies also had a strong bearing on whether participants consulted: those who had already found an effective way of controlling their headaches reported visiting their GP less often than those who struggled to contain their symptoms. Some of the respondents who were no longer seeing their GP were using over-the-counter medicines for their headaches and appeared to have them under control so did not feel they needed to consult with their GP. Other reasons for not consulting included: belief that the doctor did not have enough time; previous bad experience with a doctor; being concerned about the expense of treatments; or worry about what the doctor might think about them.

##### Own management techniques

In addition to using medicines and consulting health professionals, participants described a number of other techniques that they had found helpful for alleviating their symptoms. Common strategies included lying down in darkened rooms, or using cold or warm compresses. However, some described less conventional techniques such as digging their fingers into their necks or banging their heads off walls.

## Discussion

### Summary of findings

We found that headaches frequently adversely affect the lives of sufferers, with some wide-ranging effects on work, family life, social activities and mood. Many individuals had developed their own explanations for their headaches and many reported worry about the cause of the headaches. As a result most participants were searching for an increased understanding of their condition. Although the GP was seen as an important source of advice and treatment, many individuals were concerned about taking medication and many were trying complementary therapies or using their own management techniques.

### Strengths and limitations of the study

This study interviewed men and women from the general population who suffered from different types of headache with various causes, frequency and severity. It is one of the first qualitative studies to explore the experiences and perceptions of people with varying headache types and provides important in-depth understanding of the range of problems headache sufferers face including the impact of headaches, understanding and knowledge about headaches and experiences and expectations associated with healthcare and treatment. By studying the broad spectrum of headache types, rather than limit ourselves to one specific type, we were able to identify a range of issues relevant to headache and its impact. Although this approach means that some of our findings will not be relevant to all headache sufferers the approach has provided a useful insight into the differences in perceptions and experiences of headache sufferers. It has also highlighted some issues that will be worth exploring in sub-groups of individuals, particularly with a view to developing new treatments. Information on the cause of the head pain was not determined in the original cohort and so we do not know if the individuals interviewed in this study had a primary headache disorder or whether the headache was a result of some other cause such as trauma. Our results can only apply to the age range of individuals included in the sample. Specific issues important to groups outwith this age range will not have been identified. In addition, since participants were sampled from a group of individuals with self-reported chronic pain (pain persisting beyond three months), our findings may not reflect the experiences of people who's headaches have not persisted this long. Local availability of different services may have driven use of certain services, and this may vary by geographical location across the UK. The researcher's roles and opinions may have affected both the data collection and interpretation. This was minimised where possible by having checks on the data by a second member of the research team.

### Important issues to emerge and comparison with other studies

Several important issues emerged from our study, some of which have been reported in previous studies and some of which have not been documented before. The impact of headaches on life emerged as a key theme in this study and has been well documented in previous quantitative studies [4-11] and in the qualitative studies that have been undertaken [15-17]. Our findings on the impact of headache on daily functioning (including impact on work, social and family life and mood) are broadly in line with previous qualitative studies of headache, despite differences in the type of headache investigated and the populations studied. In a focus group study in the US with 24 people who had been experiencing migraines for at least 6 months Cottrell et al [15] reported that all aspects of family, recreational and social activities had been hampered by their headaches. The effect on work was substantial, with participants reporting having to go home early and feelings of guilt for not carrying a fair share of the workload. They also found that many participants expressed apprehension about losing their jobs because of the time they missed. This did not emerge as a clear issue in our study and may be more relevant to migraine sufferers who are likely to take more time off work. Ruiz de Velasco et al [16] in a Spanish study on quality of life in migraine patients reported similar impacts on work and studies, life within the family, social relationships, and leisure time. They found that respondents had difficulties in performing their duties at work and felt they couldn't fulfil their real potential. Many participants reported difficulties in going out with other people and some reported that they had declined to participate for fear of having a migraine attack. They found a greater impact on family relationships than we reported, particularly in terms of the effect on children. This may have been because the age range of our participants meant that fewer respondents had young children at home. Like us Ruiz de Velasco et al found that the impact on mood was an important issue with their respondents reporting mood swings, unhappiness and hopelessness and a greater lack of emotional control. Contrary to this Cottrell et al [15] reported that their participants denied emotional distress despite the emotional charge obvious in many of their statements. Our study adds some understanding to the wide-ranging and extreme effects headache can have on peoples lives, for example forcing people to change career or leading to severe depression and thoughts of suicide. Further work is necessary to identify ways that impact can be minimised and quality of life improved.

The second theme to emerge from our study was knowledge and understanding about headaches which showed that many participants had formed their own ideas about their headaches, which had often led to considerable worry, and many were searching for an increased understanding of their headaches from a variety of sources. In a qualitative study of patients decision making for migraine and chronic daily headache management Peters et al [17] reported similar issues to us. They found that an individual's knowledge about headaches was acquired through the participant's own and other people's experiences and through information gathering. Patients sometimes had to recognize the limitations of his/her own ideas for headache management and resort to GP consultation. They found that individuals had sought information from a variety of sources including health professionals, family and friends, the media and specialized organisations. Cottrell et al [15] also found that people with migraines often need more general information about migraines and migraine management. It is clear then that people are interested in understanding more about their headaches and securing relevant information about it. This desire for further information needs greater investigation. The issue of worry raised in this study has not been highlighted clearly before. Most participants in our study had, at some point, worried, often substantially, that their headaches were caused by serious pathology, even patients who had been given a formal diagnosis. The fact that participants who had received brain scans stated that their worry had reduced and in some cases their headaches had diminished as a result merits further enquiry.

The third theme to emerge focussed on issues about management of headaches. We found that most participants had used or were still using a variety of management strategies including conventional, complementary and personally developed management techniques and that satisfaction with different management strategies varied widely. Our findings on issues related to management were largely consistent with previous studies [15,17]. Cottrell et al [15] found that while some of their group members were happy with the medical care they received others reported feeling 'dismissed' by physicians who did not appear to take headaches seriously. In addition they found that headache sufferers were taking responsibility for researching new treatment options including medications and alternative treatments. Peters et al [17] found similar reasons to us for GP consultation with headache severity, patient preferences, previous experience of healthcare services and other people's experiences of health care all playing a role in management decisions. Some of the issues that emerged from our study around headache management have not been investigated in qualitative studies of headache, but are consistent with qualitative studies of individuals with other chronic conditions, particularly with respect to general practitioner involvement and medication [23-27]. For example, previous qualitative studies have clearly highlighted limitations with the GP consultation, [23-25] while other studies have found that chronic users of medication often worry about the side effects of their medication and possible drug interactions [26,27]. The full range of management techniques used by headache sufferers including unconventional techniques developed by the sufferers has not been highlighted before and this needs further investigation to optimise appropriate management of headache in the community.

### Conclusion and future implications

An in-depth understanding of the experiences of people with headache is important if we are to develop new treatment or interventions that might reduce the burden of the disorder. This study has highlighted some issues that require further investigation and provided useful information that might guide the development of such interventions. For example, the frequent wish to increase understanding of headache suggests that many people with headache could benefit from detailed information about the causes and management of headaches. Research is needed to determine whether such educational strategies result in beneficial health outcomes. Educational strategies are being developed for patients and health care professionals, particularly with reference to migraine [28,29] and will require future evaluation. Open accessing scanning may be a useful intervention to rule out serious underlying pathology and reduce anxiety in headache sufferers. Previous work has shown that a single visit to a neurological clinic for headaches not due to structural disease results in the number of attendances at general practice for headache dramatically falling [30,31]. However, numbers have been small and it may be that patients present to general practice just as often, but with different symptoms. There is little evidence that scanning is of any use in non-anxious patients [32] and it is likely therefore that the most cost effective route would be to scan only those who present with anxiety.

## Declaration of competing interests

The author(s) declare that they have no competing interests.

## Authors' contributions

DAL participated in the design of the study, carried out the interviews, conducted the analysis and drafted the manuscript. AME conceived the study, and participated in its design and coordination and helped to draft the manuscript. PCH conceived the study and participated in its design and coordination and helped to draft the manuscript. All authors read and approved the final manuscript.

## Pre-publication history

The pre-publication history for this paper can be accessed here:


